# Identification of new co-diagnostic genes for sepsis and metabolic syndrome using single-cell data analysis and machine learning algorithms

**DOI:** 10.3389/fgene.2023.1129476

**Published:** 2023-03-16

**Authors:** Linfeng Tao, Yue Zhu, Jun Liu

**Affiliations:** ^1^ Department of Critical Care Medicine, Suzhou Municipal Hospital, Suzhou Clinical Medical Center of Critical Care Medicine, The Affiliated Suzhou Hospital of Nanjing Medical University, Gusu School of Nanjing Medical University, Suzhou, China; ^2^ Department of Breast and Thyroid Surgery, The Affiliated Suzhou Hospital of Nanjing Medical University, Gusu School, Nanjing Medical University, Suzhou, China

**Keywords:** co-diagnostic, sepsis, metabolic syndrome, single-cell data analysis, machine learning algorithms

## Abstract

Sepsis, a serious inflammatory response that can be fatal, has a poorly understood pathophysiology. The Metabolic syndrome (MetS), however, is associated with many cardiometabolic risk factors, many of which are highly prevalent in adults. It has been suggested that Sepsis may be associated with MetS in several studies. Therefore, this study investigated diagnostic genes and metabolic pathways associated with both diseases. In addition to microarray data for Sepsis, PBMC single cell RNA sequencing data for Sepsis and microarray data for MetS were downloaded from the GEO database. Limma differential analysis identified 122 upregulated genes and 90 downregulated genes in Sepsis and MetS. WGCNA identified brown co-expression modules as Sepsis and MetS core modules. Two machine learning algorithms, RF and LASSO, were used to screen seven candidate genes, namely, STOM, BATF, CASP4, MAP3K14, MT1F, CFLAR and UROD, all with an AUC greater than 0.9. XGBoost assessed the co-diagnostic efficacy of Hub genes in Sepsis and MetS. The immune infiltration results show that Hub genes were expressed at high levels in all immune cells. After performing Seurat analysis on PBMC from normal and Sepsis patients, six immune subpopulations were identified. The metabolic pathways of each cell were scored and visualized using ssGSEA, and the results show that CFLAR plays an important role in the glycolytic pathway. Our study identified seven Hub genes that serve as co-diagnostic markers for Sepsis and MetS and revealed that diagnostic genes play an important role in immune cell metabolic pathway.

## Introduction

Sepsis is caused by dysregulation of the immune system’s response to infection (Font et al., 2020). In spite of a growing number of innovative treatments, Sepsis still ranks as a leading cause of death in hospitals. As a result of the diversity of clinical symptoms of Sepsis, diagnosing, treating, and managing patients with Sepsis remains challenging (Huang et al., 2019). This is why it is urgent to understand the pathophysiology of Sepsis in order to identify new biomarkers that may improve the diagnosis, treatment, and prognosis of the disease.

The prevalence of Metabolic syndrome (MetS) has also been rising dramatically in adults in contrast to the prevalence of Sepsis (Grundy et al., 2004). MetS is an umbrella term for various cardiovascular disease risk factors such as diabetes, obesity, and hypertension, the mechanisms of which are not yet fully understood (Pugazhenthi, 2017). There has been some evidence that MetS increases the risk of adverse outcomes, including coronary artery disease, which can result in a higher mortality rate in patients with Sepsis (Grundy et al., 2004; Wilson et al., 2005). Sepsis is a primarily acute inflammatory condition with mortality peaking within days, whereas MetS-related complications manifest as chronic inflammation that results in mortality over several years (Meydan et al., 2018). It has been shown that both Sepsis and MetS are inflammation-related diseases. A deeper understanding of the emerging long non-coding RNAs (lncRNAs) has revealed the influence of inflammation-related molecular agents and cytokines in both Sepsis and MetS (Meydan et al., 2018). Linking non-coding RNA regulators to Sepsis and MetS may lead to the identification of new high-value biomarkers as well as targets for clinical intervention (Meydan et al., 2018). It has been reported that Sepsis and MetS are regulated by the same upstream regulators, such as microRNAs (miRNAs) and lncRNAs (Meydan et al., 2018). In MetS, Lethe lncRNAs are known to inhibit the binding of NF-κB’s p65 subunits to DNA, thus exerting anti-inflammatory effects by inhibiting NF-κB’s DNA binding, which could have a beneficial effect on Sepsis-induced immune disorders (Zgheib et al., 2017). HOTAIR is another lncRNA involved in pathogen inflammation through the NF-κB pathway (Wu et al., 2016). HOTAIR lncRNA-associated transcripts are overexpressed in adipose tissue, where they play an important role in the Metabolic syndrome (Wu et al., 2016). In an analysis based on human peripheral RNA sequencing data, 1,152 acutely ill patients were recruited for this study and divided into systemic inflammatory response syndrome (SIRS) and four Sepsis groups of increasing severity, with HOTAIR-related genes elevated 2-3-fold in patients with severe Sepsis compared to SIRS (Meydan et al., 2018). These studies suggest that HOTAIR plays a significantly interactive role in Sepsis and MetS. Recent findings have demonstrated that MetS improved survival in septic mice, attenuated the increase in plasma nitric oxide (NO) in septic mice, and lower NO production may help reduce hypotensive events in the MetS animal group (Nakama et al., 2021). These findings provide new insights into the association between MetS and Sepsis in mice. Although the relationship between the two diseases is supported by some evidence, the molecular mechanisms shared by Sepsis and MetS are still being explored.

More recently, bioinformatics has been widely applied to oncological and non-oncological diseases, including sepsis (Lai et al., 2020; Li Z. et al., 2021; Wu Z. et al., 2021). Previous studies have focused on differential genes in the blood of Sepsis patients and revealed the molecular pathways of differential genes (Chen et al., 2021). However, the common diagnostic genes of Sepsis and MetS and the shared metabolic pathways remain unclear. Therefore, in this study, WGCNA was utilized to identify the common pivotal genes in the plasma of Sepsis patients and MetS. In addition, the CIBERSORT algorithm was used to identify immune infiltrating cells and to investigate the potential mechanism of the Hub gene in immune cells, which will provide some guidance for the identification of common biomarkers for Sepsis and MetS in the future and provide a theoretical basis for new diagnosis and treatment.

## Materials and methods

### Identification and analysis of differential genes

We obtained a sepsis RNA microarray dataset and a sepsis RNA-seq dataset [GSE28750, GSE154918], respectively (Sutherland et al., 2011; Herwanto et al., 2021). Among them, there were 30 samples (20 normal and 10 sepsis) in the GSE28750 dataset. In the GSE154918 dataset, 60 samples (40 normal and 20 sepsis) were selected. Two samples of peripheral blood mononuclear cells (PBMCs) with sepsis and two samples of normal PBMCs were selected in the GSE167363 dataset (Qiu et al., 2021) from GEO database (Barrett et al., 2013); we also obtained the MetS dataset GSE98895 (D'Amore et al., 2018). Forty samples (20 normal and 20 MetS) were selected in the GSE98895 dataset. In order to analyze the data, the software R was employed. Principal component analysis (PCA) was used before and after correcting batch effects and visualizing the distribution of these datasets. The datasets were corrected for background, transformed by log2, and normalized. We also merged the datasets GSE28750 and GSE154918, and used the Combat method in the “sva” package to batch-correct the merged data (Leek et al., 2012). Then, the merged result was viewed by PCA dimensionality reduction algorithm. Following the identification of differentially expressed genes (DEGs) by the limma analysis, the following filtering criteria was applied to screen for significantly differentiating genes: *p* < 0.05 and |log2 Fold change (FC) | > 1.0 (Ritchie et al., 2015). Finally, Venn plots were used to illustrate common genes which are up- and downregulated in Sepsis and MetS, respectively. Meanwhile, Gene Ontology (GO) enrichment analysis of the common DEGs were conducted utilizing the “clusterProfiler” package of R software (Wu T. et al., 2021). The pathways with *p* < 0.05 were considered significant.

### Analysis of weighted gene Co-expression networks

The “WGCNA” R package was employed to identify genes associated with Sepsis and MetS using a weighted gene co-expression network (Langfelder and Horvath, 2008). We used genes with expression >0 for further analysis to exclude outlier data. For the construction of the co-expression network, co-expression analysis was utilized. Flash clust was used for cluster analysis in our study. Clustering each sample from the beginning ensured the reliability of the network. As a result of calculating Pearson product moment correlation coefficients between gene pairs, we group genes with similar expression patterns into modules, thus creating a correlation matrix. Soft threshold functions are also used to transform the correlation matrix into a weighted adjacency matrix. To identify the most relevant Sepsis and MetS modules, we set the optimal soft threshold and identified the multi-co-expressed module genes simultaneously.

### Screening candidate genes with machine learning

In order to further filter candidate genes for Sepsis and MetS diagnosis, two machine learning algorithms have been applied: random forest (RF) (Garge et al., 2013) and least absolute shrinkage selection (LASSO) (Alhamzawi and Ali, 2018). The search for important genes was carried out using the “random forest” R package. Based on decision tree theory, the RF algorithm was classified according to its ability to handle high-dimensional data and select highly informative gene clusters. The RF algorithm was used to screen diagnostic genes whose importance score exceeded 0.5. LASSO regression can be used for high-dimensional data to enhance the effectiveness of the analysis. For further reducing the dimensions of the obtained genes, the LASSO algorithm was applied to obtain the final diagnostic genes. The genes deemed most significant were selected as the core genes for further research.

### Assessing the diagnostic value of candidate genes

In supervised integrated learning, eXtreme Gradient Boosting (XGBoost) is one of the most commonly used algorithms due to its scalability and convenience (Parente, 2021). For the model, hyperparameters were tuned using an optimisation method based on a Bayesian sequence model. Optimisation was performed on the training set, using K-fold cross-validation (K = 10) for continuous iteration. Candidate gene models were constructed using XGBoost on the training data set (GSE154918) and evaluated on the validation data set (GSE28750). Following that, the diagnostic efficacy of the model was evaluated using receiver operating characteristic curves (ROC), precision-recall curves (PR), and areas under the curve (AUC). This was verified in MetS patients.

### Investigating the infiltration of immune cells

Assess the presence of immune cells in each sample using the CIBERSORT algorithm (Hu et al., 2022). Based on linear support vector regression, the CIBERSORT deconvolution algorithm calculates the percentage of 22 immune cells in tissues or cells using machine learning. These 22 cell types included dendritic cells, CD4^+^ and CD8^+^ T Cells, B cells, macrophages M1 and M2, monocytes, neutrophils, natural killer cells, and natural killer cells. The proportion of immune cells in peripheral blood mononuclear cells (PBMCs) was compared between the disease and control groups. Meanwhile, the relationship between the Hub gene and immune cells in Sepsis and MetS was explored.

### Single-cell data analysis

To analyze the dataset of single cells GSE167363, we used the “Seurat” R package to run PCA and t-distributed stochastic neighbor embedding (t-SNE) (Butler et al., 2018). Those cells with more than 4,000 features, mitochondrial genes over 25%, or less than 200 features were excluded from the analysis. After scaling the level of gene expression, a technique called “LogNormalize” was utilized to normalize the data. After normalizing the data, 3,000 highly variable genes (HVGs) within each sample were identified using the “vst” method. Principal component analysis (PCA) was then performed and the significant principal components (PCs) were identified using the elbow method. In the end, t-SNE analysis was performed using 20 PCs that were chosen. We used FindClusters function to cluster cells into 21 clusters. In order to locate differentially expressed genes (DEGs) for each cluster, the logfc. threshold parameter was set to 0.25 using the FindeMarker function. The “singleR” package comes with seven reference datasets, of which 5 are human and 2 are mouse, and we have selected “HumanPrimaryCellAtlasData” as the reference dataset (Aran et al., 2019). An automated annotation using “SingleR”package in conjunction with DEGs in each cluster to identify cell types, and then identifying the cell types in each cluster separately (Li et al., 2020). The Hub gene expression was also visualized by violin diagrams in different immune cells.

### Correlation of single-cell metabolic pathways with core genes

Molecular Signature Database (MSigDB) (Li J. et al., 2021) was used to download the hallmark gene set, and single sample gene set enrichment analysis (ssGSEA) was done to analyze metabolic pathways associated with the Hub genes. Lastly, we analyzed the correlation between immune cells and metabolism in Sepsis and MetS. Use the Pearson correlation method in the “stats” package of R language to calculate its correlation. Differences in metabolic pathway scores between single cell subpopulations were demonstrated by violin plots, where significant differences were determined by Wilcoxon tests.

### Statistical analysis

All statistical tests were performed using R version 4.1.2. The Wilcoxon or Student’s t-test was used to analyse the difference between the two groups. Correlations between variables were determined using Pearson’s or Spearman’s correlation test. Statistical significance was set at a two-tailed *p* < 0.05.

## Results

### Screening of common differential genes

**FIGURE 1:**
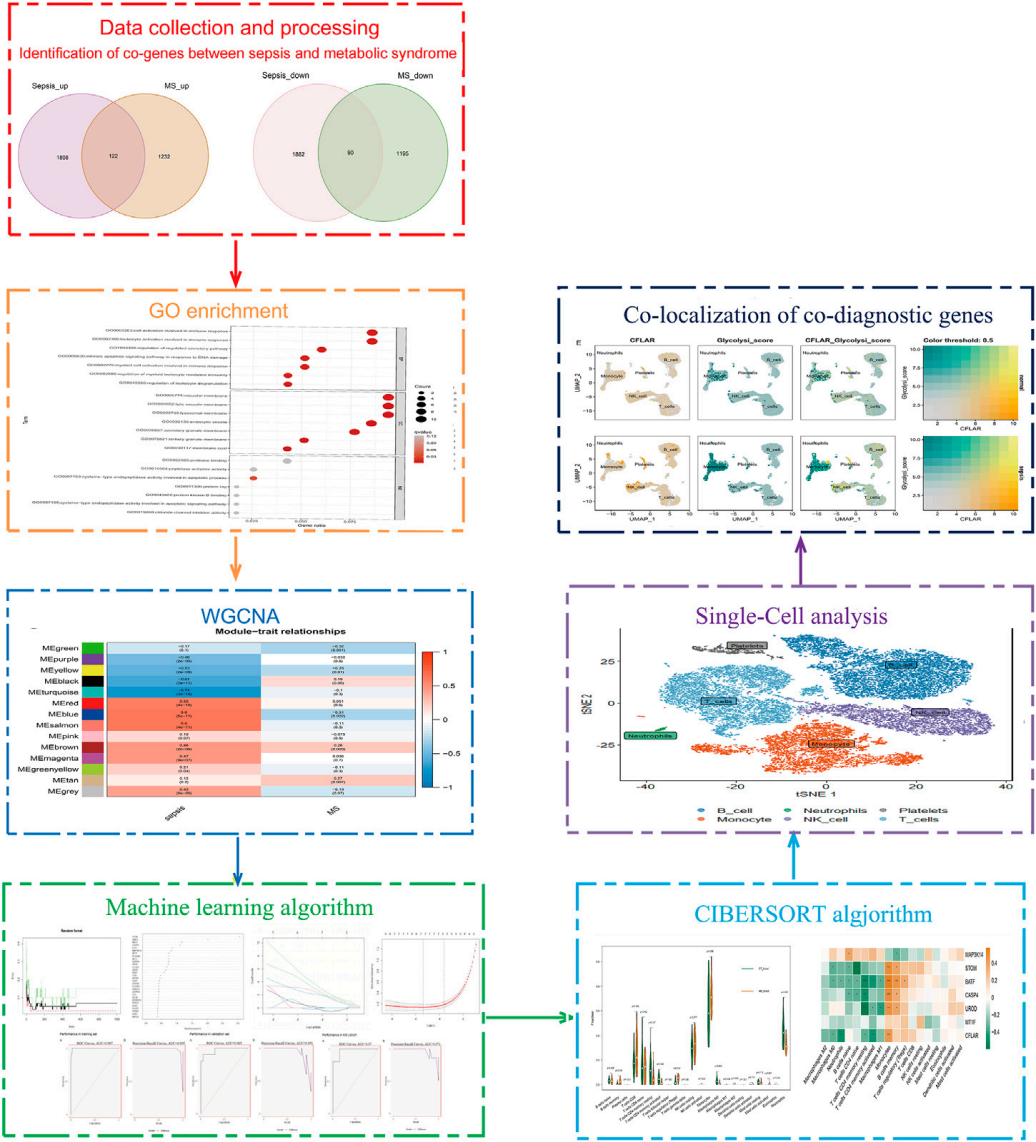
Research technology flow chart.

**FIGURE 2:**
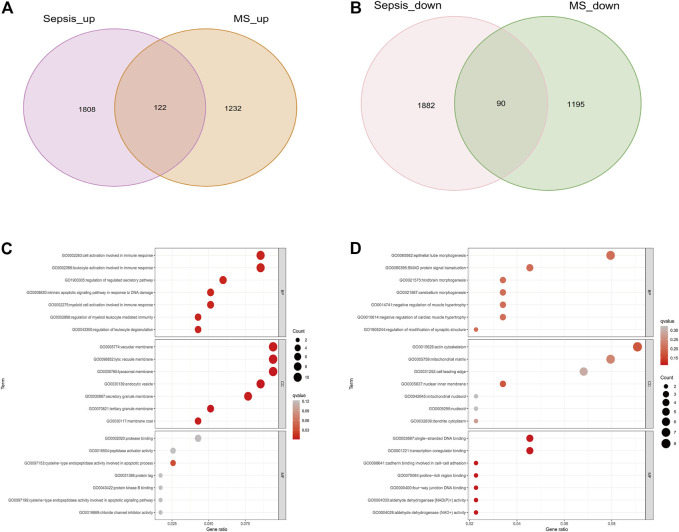
Differential analysis and KEGG enrichment analysis of Sepsis and MetS patients **(A)** Intersection of DEGs upregulated by sepsis and DEGs upregulated by MetS **(B)** The intersection of MetS downregulated DEGs and Sepsis downregulated DEGs **(C)** GO enrichment analysis for common genes upregulated **(D)** Analysis of downregulated common genes based on GO enrichment.

As shown in Figure 1, the study flow chart explains how it was conducted. The PCA was performed on three datasets (GSE28750, GSE154918 and GSE98895) before corrections and normalizations (Supplementary Figures S1A, B). The datasets were normalized, and 3,902 DEGs (1930 upregulated and 1972 downregulated) were found in Sepsis, while 2,639 DEGs (1,354 upregulated and 1,285 downregulated) were found in MetS. By identifying common DEGs between Sepsis and MetS, 122 common upregulated DEGs and 90 common downregulated DEGs were found (Figures 2A, B) (Supplementary Tables S1, S2). GO enrichment analysis of the identified common DEGs was performed to investigate their biological functions and pathways. According to GO analysis, the commonly upregulated DEGs are mainly involved in cell activation and leukocyte activation involved in immune response and regulation of regulated secretory pathway, while the common downregulated DEGs were enriched in epithelial tube morphogenesis, actin cytoskeleton, mitochondrial matrix, SMAD protein signal transduction (Figures 2C, D) (Supplementary Tables S3,S4).

### Analysis of Co-expressed gene modules in WGCNA

**FIGURE 3:**
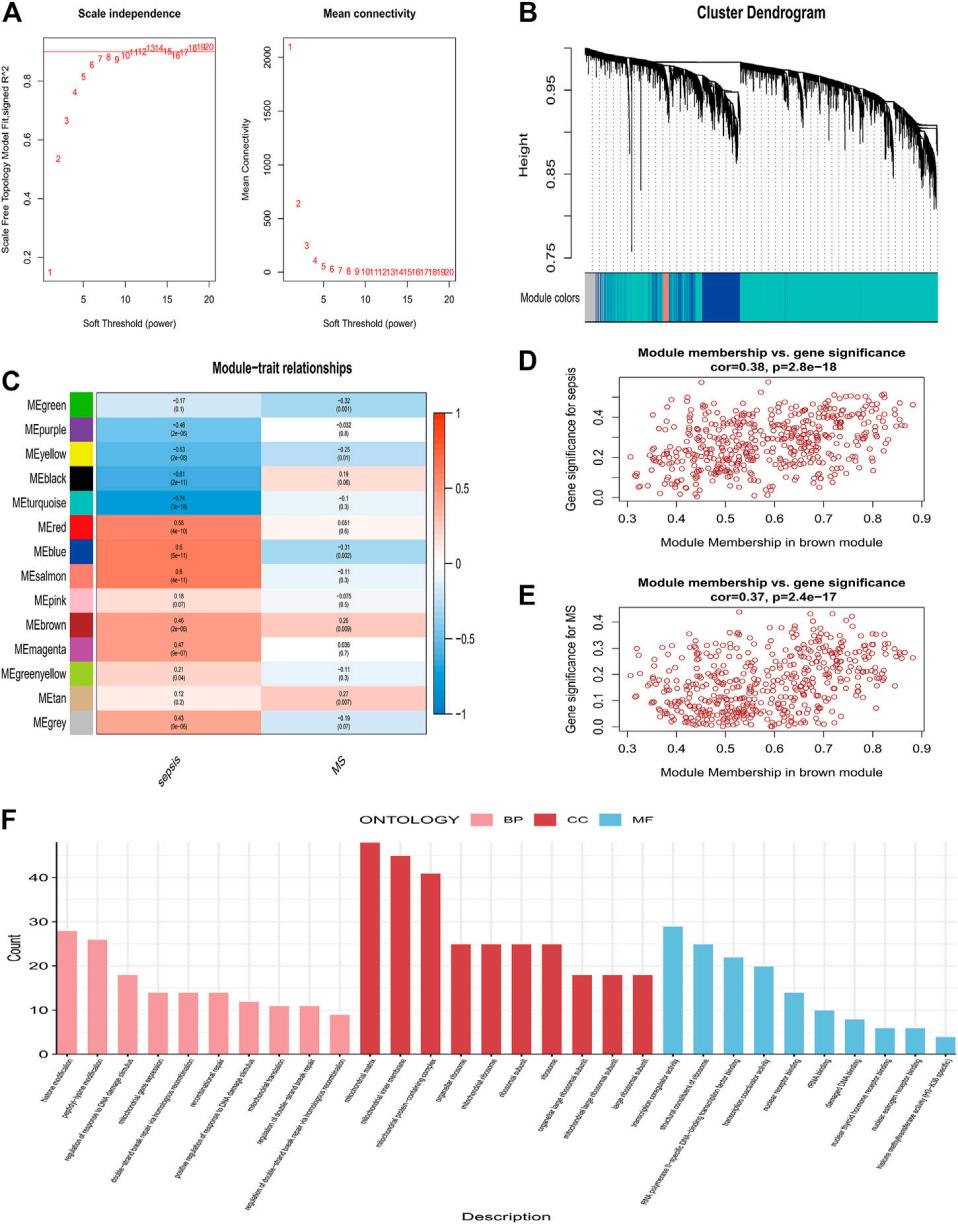
Co-expression modules and enrichment analysis in patients with Sepsis and MetS **(A)** Analysis of the network topology of soft threshold power **(B)** Cluster dendrogram identifying co-expressed genes in Sepsis and MetS **(C)** The module–trait relationships in Sepsis and MetS. Correlations and *p*-values are provided for each module **(D)** Correlation of brown modules with Sepsis **(E)** Correlation between brown modules and MetS **(F)** Analysis of GO enrichment for brown module genes.

With a threshold of 80, 2 outlier samples were detected and removed, and 98 samples were retained (Supplementary Figures S1C, D). The “pick Soft Threshold” function of the “WGCNA” package is used to filter out power parameters from 1 to 30. As a soft threshold, a power of 6 was selected for ensuring the scale-free network (Figure 3A). A total of 14 modules containing genes with similar co-expression traits were obtained using the “cutree” dynamic and module eigengenes functions (Figure 3B). The heatmap displayed the correlation between each module and the diseases (Figure 3C). “Brown” modules indicate that Sepsis and MetS are highly linked (Sepsis: r = 0.46, *p* = 0.009; MetS: r = 0.26, *p* = 0.003). Sepsis and MetS have positively linked genes in the brown module (Sepsis: cor = 0.38, *p* = 2.8e-18; MetS: cor = 0.37, *p* = 2.4e-17) (Figures 3D,E). For this brown module gene, a GO analysis was performed. The results show that it was mainly enriched in histone modification, peptidyl−lysine modification, regulation of response to DNA damage stimulus in biological process (BP), Mitochondrial matrix, mitochondrial inner membrane and Mitochondria containing protein complexes in cellular component (CC) and transcription coregulator activity and structural constituent of ribosome in molecular function (MF) (Figure 3F).

### Identification of candidate central genes using machine learning

**FIGURE 4:**
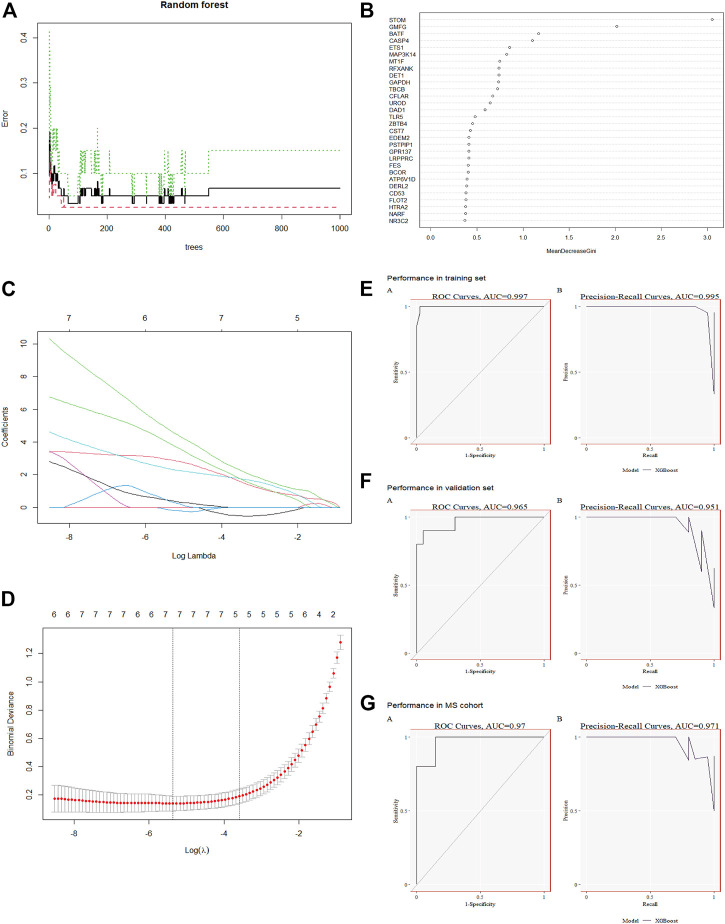
Co-diagnostic gene screening and model construction using machine learning **(A)** Relationship between the number of decision trees and the error rate. The yellow node represents the root node, the black node represents the non-leaf node, and the red leaf node represents the classification result **(B)** The top 40 candidate genes screened by random forest. Gene importance coefficients are indicated by the horizontal coordinates. The vertical coordinates indicate the names of the genes **(C)** Spectrum of Lasso coefficients for candidate genes **(D)** Evaluation of the optimal tuning parameters log(Lambda) in LASSO regression with cross-validation **(E)** A training set for XGBost modeling has been created for Sepsis **(F)** Demonstration of validity using the Sepsis Validation Set **(G)** MetS dataset validation.

We used the RF algorithm in combination with LASSO regression to finally obtain seven diagnostic genes, including STOM, BATF, CASP4, MAP3K14, MT1F, CFLAR, UROD (Figures 4A–D). Afterwards, we evaluated these genes’ diagnostic value. The AUC values of ROC curves were 0.995 of STOM (Supplementary Figure S2A), 0.996 of BATF (Supplementary Figure S2B),0.995 of CASP4 (Supplementary Figure S2C), 0.995 of MAP3K14 (Supplementary Figure S2D), 0.968 of MT1F (Supplementary Figure S2E), 0.934 of CFLAR (Supplementary Figure S2E), 0.973 of UROD (Supplementary Figure S2E). All seven gene features had high accuracy with AUC >0.9, demonstrating their predictive power. Based on the training set GSE154918, we constructed a candidate gene model (STOM, BATF, CASP4, MAP3K14, MT1F, CFLAR) and evaluated it on the validation set GSE28750. As displayed in Figure 4E, in GSE154918, the AUC of ROC value was 0.997 and the PR value was 0.995. The ROC and PR values for GSE28750 are 0.965 and 0.951, respectively (Figure 4F), demonstrating the model’s diagnostic accuracy. It has also been validated in MetS, indicating that the model is applicable and effective in MetS, with a ROC of 0.97 and a PR of 0.971 (Figure 4G).

### Infiltration of immune cells in sepsis and MetS patients

**FIGURE 5:**
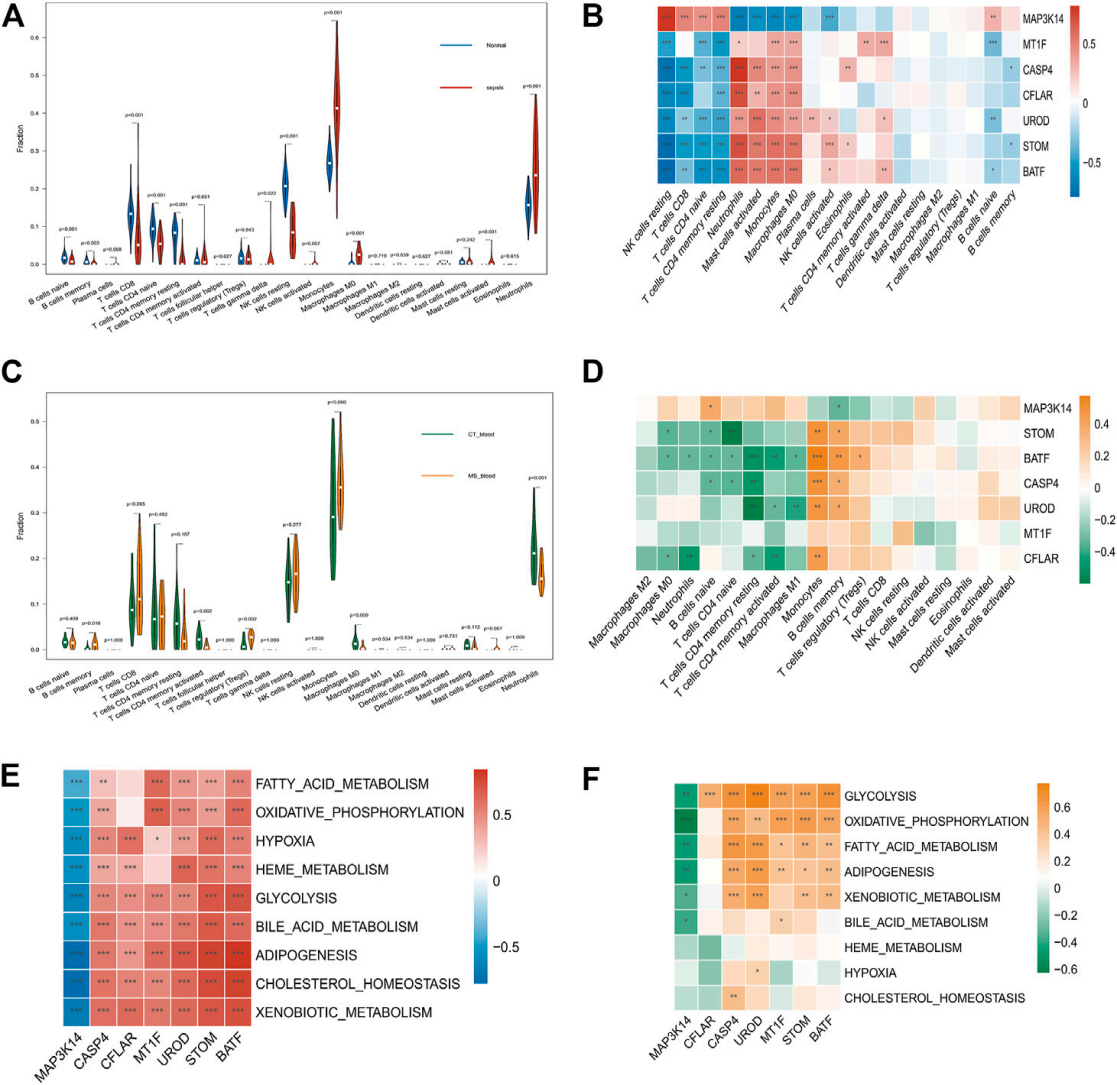
Immune cells and metabolic pathways in patients with Sepsis and MetS **(A)** Infiltration of immune cells between Sepsis and healthy samples **(B)** Immune infiltration analysis of seven candidate genes in Sepsis **(C)** Comparison of immune cell infiltration between samples from the MetS group and the normal group **(D)** Analysis of seven MetS candidate genes’ immune infiltration **(E)** Correlation between the expression levels of seven hub genes and the ssGSEA enrichment scores of the classical metabolic pathways in the sepsis data **(F)** Correlation between the expression levels of seven hub genes and the ssGSEA enrichment scores of the classical metabolic pathways in the MetS data. *p < 0.05, **p < 0.01, ****p < 0.001.

Sepsis and MetS patients with immune infiltration were studied. In addition, heat maps show the differential expression of seven key genes in immune cells (Figures 5B, D). Normal tissues contained fewer neutrophils and monocytes than Sepsis tissues (*p* < 0.05). A comparison of Sepsis tissues and normal tissues revealed that Sepsis tissues contained significantly fewer naïve B cells, memory naïve B cells, CD8 naïve T Cells, and CD4 naïve T Cells (Figure 5A). The expression of STOM, BATF, CASP4, MT1F, CFLAR, and UROD was negatively correlated with infiltration levels of resting NK cells, CD4 naïve T Cells, CD8 T Cells, and CD4 resting T Cells. And MAP3K14 expression was negatively associated with neutrophils, activated mast cells, monocytes, macrophage M0, and NK activated cells (Figure 5B). We also calculated the immune cell content in patients with MetS, and monocyte proportions were higher in patients with MetS than in controls (Figure 5C). The expression levels of key genes differed significantly in patients with MetS, with six genes (STOM, BATF, CASP4, MT1F, CFLAR, UROD) being expressed at lower levels in M0, M1 and M2 macrophages than in MAP3K14. However, six genes (STOM, BATF, CASP4, MT1F, CFLAR, UROD) were expressed at higher levels in monocytes, B memory cells and T cell regulation (Tregs), but all higher than MAP3K14 (*p* < 0.05) (Figure 5D). To explore the potential metabolic pathways involved in hub genes, correlations between hub genes and classical metabolic pathways were analysed. In sepsis samples, significant positive correlations were found between six hub genes (STOM, BATF, CASP4, MT1F, CFLAR, UROD) and the pathways of glycolysis, bile acid metabolism, adipogenesis, cholesterol homeostasis and xenobiotic metabolism, while the pathways of glycolysis, bile acid metabolism, adipogenesis, cholesterol homeostasis and xenobiotic metabolism showed negative correlations with MAP3K14 (Figure 5E). Similarly, MetS samples expressing six central genes (STOM, BATF, CASP4, MT1F, CFLAR, UROD) showed positive correlations in glycolysis, oxidative phosphorylation and fatty acid metabolism pathways (Figure 5F), while MAP3K14 expression was negatively correlated with glycolysis, oxidative phosphorylation and fatty acid metabolism (Figure 5F).

### Single-cell sequencing analysis in sepsis and normal patients

**FIGURE 6:**
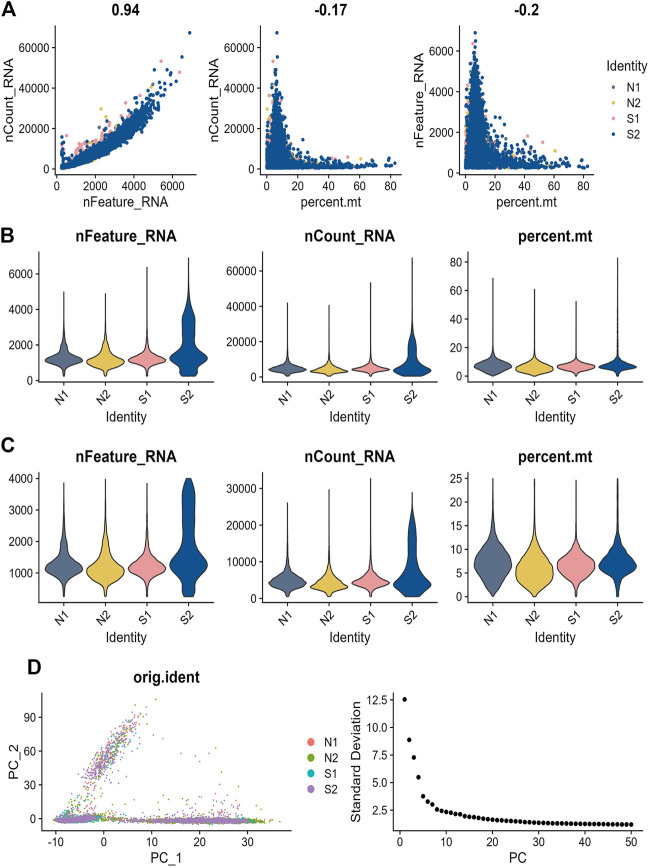
Sepsis single cell data quality control process **(A)** Analysis of the correlation between gene expression and cell counts and mitochondrial content in each sample. **(B)** Precent. mt, nFeatureRNA, and nCountRNA for each sample before filtering **(C)** nCount RNA, nFeature RNA, and precent. mt for each sample after filtration **(D)** Principal component analysis (PCA) plot, where each dot in the plot, represents a cell. Elbow plot, a method used to determine the number of PCs.

**FIGURE 7:**
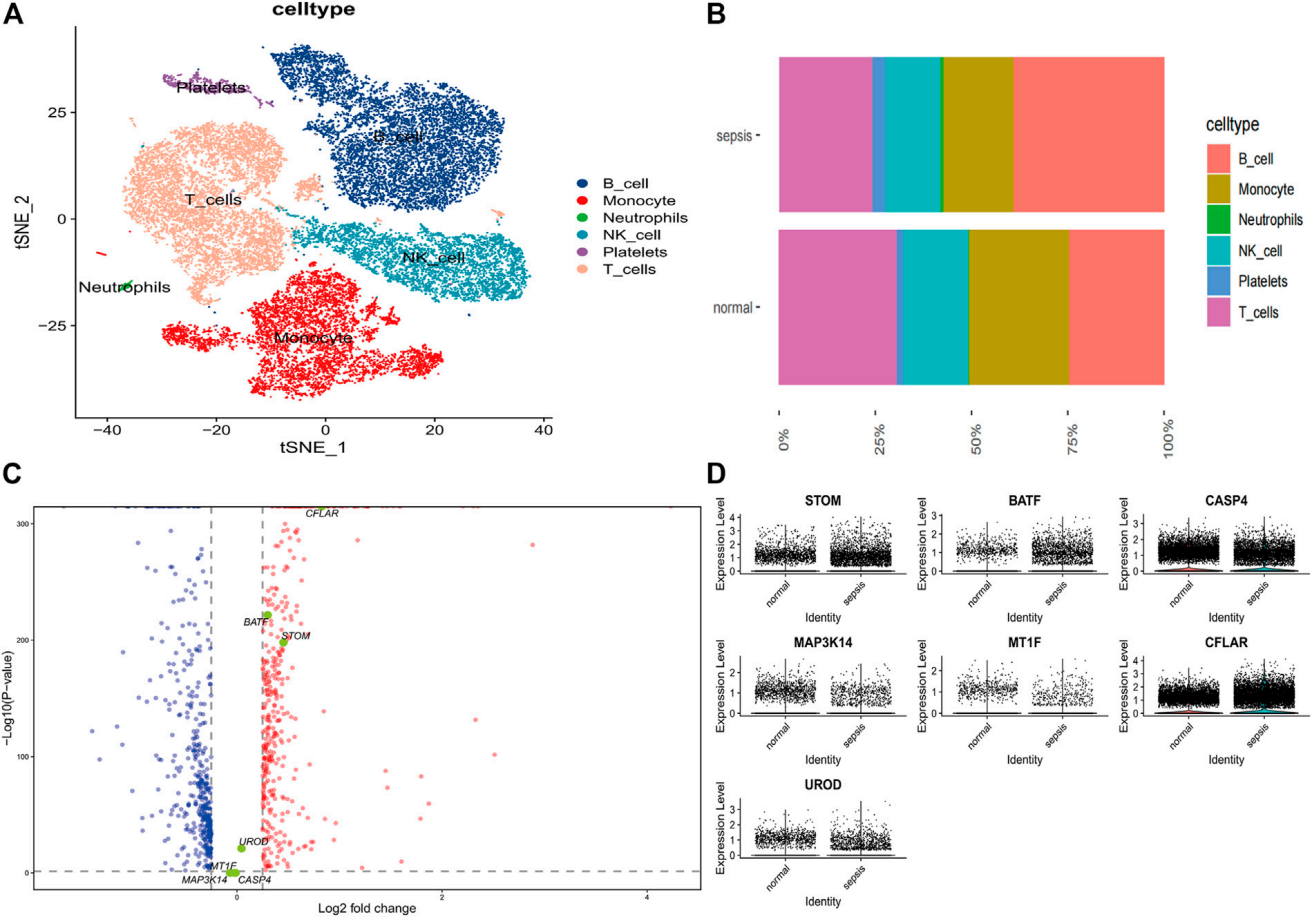
Single-cell subpopulation identification and expression levels of diagnostic genes in Sepsis and normal groups **(A)** TSNE display plot of cell subpopulations in Sepsis patients **(B)** Comparison of immune cell composition in patients with Sepsis and normal **(C)** Volcano diagram showing differential genes, with red dots representing upregulated genes, blue dots representing downregulated genes and yellow dots representing hub genes **(D)** An expression plot showing the levels of diagnostic genes in Sepsis patients and normal individuals.

In order to check the quality of the single-cell dataset GSE167363, a preliminary quality check was performed. The correlation between nFeature RNA, nCount RNA, and precent. mt was examined to make sure the cell samples used in the study were of high quality. Figure 6A exhibited a positive correlation between nCount RNA and nFeature RNA representing unique molecular identifiers, with a correlation coefficient of 0.94. We excluded some cells and the result was diaplayed in Figures 6B,C. In the scRNA-seq dataset, a total of 3,000 genes with high levels of variation were identified, and ten of the most significant markers were tagged. A PCA analysis of the top 20 PCs was performed (Figure 6D). The t-SNE algorithm was used to cluster cells, obtaining 21 clusters (Figure 7A). We showed the top ten marker genes for the 21 clusters (Supplementary Table S5). In the Sepsis group, monocyte clusters, T Cells, and NK cells were decreased, and the B cell subpopulation increased (Figure 7B). We extracted mainly monocytes, NK cells, T Cells and B cells from the sepsis single cell dataset. GO enrichment analysis was performed to obtain the pathways of the differential genes (Supplementary Table S6-9) (Supplementary Figure S3-6). We show differential genes by plotting volcanoes, where red dots represent upregulated genes, blue dots represent downregulated genes, and yellow dots represent the seven core genes (Figure 7C). We found the most significant differences in CFLAR, STOM and BATF. Similarly, we compared the expression levels of the seven core genes in normal subjects and sepsis patients. In both groups, STOM, CASP4 and CFLAR were expressed at higher levels, while the remaining four genes were expressed at lower levels (Figure 7D).

**FIGURE 8:**
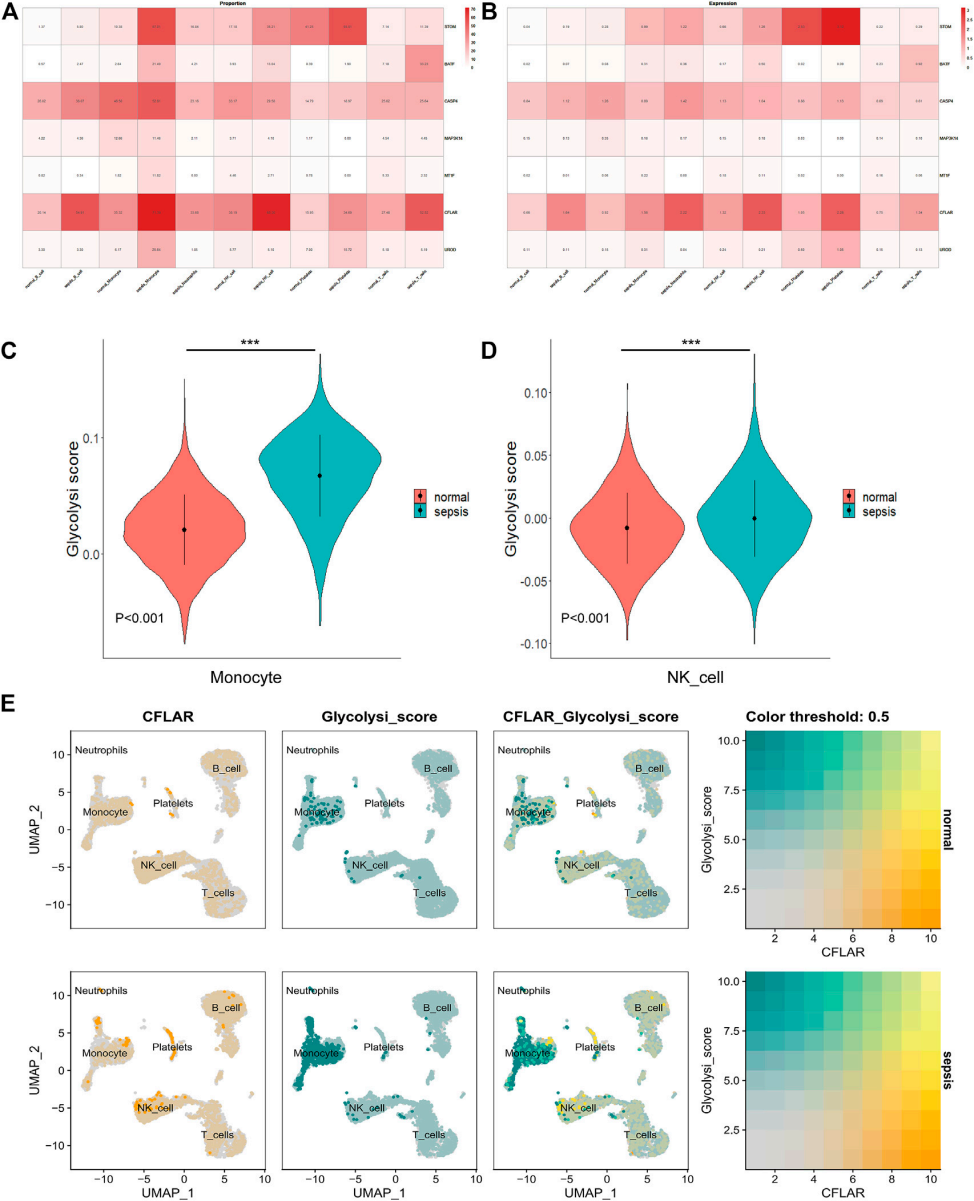
Co-localization and differential expression of co-diagnostic genes in immune cells of Sepsis patients **(A)** Co-diagnostic gene expression ratios in Sepsis and normal immune cells **(B)** Expression of co-diagnostic genes in Sepsis and normal individuals in each immune cell **(C)** A violin plot showing the difference between normal and Sepsis monocyte glucose metabolism **(D)** A violin plot depicting the differences in glucose metabolism in normal and Sepsis NK cells **(E)** Co-localization of glucose metabolic pathway and CFLAR in patients with Sepsis and healthy individuals. *** (*p* < 0.001).

A heatmap showing the proportion of core genes expressed in immune cells is then displayed. A high expression level of STOM, BATF, CASP4, and CLFAR was found in all samples in all 6 cell types (Figure 8A), and CLFAR and STOM were expressed at a high level at the gene expression level as well. STOM was highly expressed in platelet subpopulations in the normal and Sepsis groups (Figure 8B), while CLFAR expression was higher in the monocyte subpopulation, neutrophil subpopulation, and NK cell subpopulations in the Sepsis group compared to the normal group. We found that these results were generally consistent with what we found in Figure 6D in our previous analysis. Moreover, performing ssGSEA metabolic pathway analysis, we found that the glucose metabolism scores of monocytes and NK cells were different in normal and Sepsis (Figures 8C, D). Sepsis patients had higher glucose metabolism scores on monocytes and NK cells than normal patients, and CLFAR appears to be involved in this pathway (Figure 8E).

## Discussion

Bioinformatic tools and software have advanced rapidly in recent years, making public databases an excellent resource for understanding the pathophysiology of Sepsis. Sepsis mainly consists of two cross-developing pathophysiological phases, starting with immune activation and ending with chronic immunosuppression, which eventually leads to immune cell death (Nedeva, 2021). As a result, there is a tremendous amount of pro- and anti-inflammatory mediators produced, which can both lead to a severe imbalance in the immune system, as well as metabolic disorders (Hirasawa et al., 2009; van der Poll et al., 2021). The metabolic changes that cause hyperglycemia include muscle glycolysis and lipolysis, followed by hepatic glycogenesis and glycolysis (Hirasawa et al., 2009; Ferreira et al., 2022). Among patients with Sepsis, variability in blood glucose levels is associated with higher mortality rates (Ali et al., 2008; Lu et al., 2022). In addition to insulin resistance, metabolic dysfunction is also correlated with the MetS (Marette et al., 2014). The combination of WGCNA and machine learning enabled us to identify key genes common to both Sepsis and MetS, thus making it easier to identify patients at an early stage of the disease. ssGSEA was also used to assess patients’ glucose metabolism levels in order to identify metabolic disorders as early as possible.

There are a number of studies examining the relationship between Sepsis and MetS, but few have examined the diagnostic genes and metabolic pathways and immune cells that are associated with both diseases. This core brown module was developed using WGCNA, and enriched for analysis in mitochondrial matrix, endosomes, and protein pathways. The mitochondria played a key role in the production of ATP, the release of reactive oxygen species, and the regulation of cell death (Stanzani et al., 2019). Several studies have suggested mitochondrial dysfunction plays a crucial role in Sepsis-induced organ failure (Stanzani et al., 2019). Several studies suggest mitochondrial nitric oxide synthase (NOS) plays an important role in Sepsis progression, but their exact role remains unclear (Mantzarlis et al., 2017). However, mitochondrial respiratory impairment is a key factor in multi-organ failure and death in Sepsis patients (Mantzarlis et al., 2017; Stanzani et al., 2019). These studies are consistent with our findings. The study also found that Sepsis and MetS share common diagnostic genes. Based on RF and LASSO machine learning methods, seven common diagnoses were identified, including STOM, BATF, CASP4, MAP3K14, MT1F, CFLAR, and UROD. By analyzing their ROC curves, their predictive ability was demonstrated. XGBoost machine learning model was used to validate seven genes in Sepsis and MetS. Based on immune infiltration and metabolic pathway analysis, the seven genes were highly expressed at different levels of immune cell subpopulations and metabolic pathways. According to Sepsis single cell data, two genes, CFLAR and CASP4, were highly expressed in all immune cell subpopulations. CFLAR, also known as cFlip, includes CASP8 and FADD-like apoptosis regulators (Xiao et al., 2012; Faiz et al., 2018). CFLAR is an integral component of the body’s natural immune defense system (Schattenberg et al., 2011). CFLAR also plays a key role in inflammation and apoptosis in the body (Xiaohong et al., 2019). There is evidence that reduced levels of CFLAR contribute to inflammation after myocardial infarction (Xiao et al., 2012). The CFLAR was primarily found on neutrophils in immune infiltration analysis. Single-cell RNA sequencing has also revealed CFLAR expression on neutrophils in Sepsis patients’ blood. Cystein recruitment domains (CARDs) at the N terminus of CASP4 distinguish it from other cysteine-aspartate proteases (Papoff et al., 2018). In the innate immune response, CASP4 promotes phagosome-lysosome fusion, as well as maturation and secretion of pro-inflammatory molecules (Papoff et al., 2018). Apoptosis induced by endoplasmic reticulum stress (ER) is also mediated by CASP4 (Songane et al., 2018). In Sepsis and MetS, metabolic correlation analysis demonstrated the relevance of core genes in glucose metabolic pathways. As a result of our findings, we believe the common diagnostic genes we obtained contribute to the onset and progression of Sepsis and MetS. As a result of the limited number of studies that have been conducted on these two genes in Sepsis and MetS, we can only use our analysis as a preliminary reference, and further tests are necessary to confirm our findings.

Sepsis is a life-threatening organ dysfunction caused by a dysregulated host response to infection, with impaired glucose metabolism being a common problem leading to increased mortality in sepsis patients (Ferreira et al., 2022). Immune cells in patients with sepsis initially exhibit a hyperinflammatory state, which may be followed by a state of immune tolerance (Arts et al., 2017). The glycolytic pathway has been shown to be upregulated in hyperinflammatory cells, whereas the glycolytic pathway is usually downregulated in immune-tolerant cells (Arts et al., 2017). A large body of evidence suggests that changes in cellular metabolism during the inflammatory and suppressive phases of disease may influence the immune response to sepsis. We have used metabolic correlation analysis to demonstrate the association of hub genes in the glucose pathway in sepsis and MetS. Next, we will continue to investigate the mechanisms of glucose metabolism in sepsis and MetS in animal models.

Further screening by multiple machine learning algorithms yielded seven diagnostic genes common to both Sepsis and MetS, and assessment of diagnostic gene expression levels in immune cell subpopulations and metabolic pathways, all of which contribute to early diagnosis of patients. However, this study still has limitations. In order to better understand the potential of key genes in the diagnosis of Sepsis, we plan to conduct a prospective cohort study. While we have improved the diagnostic efficacy of core genes through WGCNA combined with machine learning algorithms and validated their differential expression in a single-cell dataset, we will also investigate the potential of signature genes in the treatment of Sepsis. Several genes will also be knocked out in rat models for further study.

## Conclusion

The effector genes involved in Sepsis and MetS are identified using a combination of single cell analysis and WGCNA as well as machine learning techniques. Additionally, it was found that disease diagnostic genes are associated with multiple immune cells and metabolic pathways. It is possible that glucose metabolism-related pathways are common to both Sepsis and MetS, and in Sepsis patients, glucose metabolism may work through monocytes and NK cells. We found that the CFLAR gene is likely to play a key role in glucose metabolism in Sepsis patients. This study may provide a new approach to diagnosing and treating Sepsis.

## Data Availability

The datasets presented in this study can be found in online repositories. The names of the repository/repositories and accession number(s) can be found in the article/Supplementary Material.

## Supplementary material

SUPPLEMENTARY FIGURE S1 Dataset quality control. **(A–B)** PCA between integrated 512 datasets after de-batching. **(C-D)** Before and after clustering of WGCNA analysis

SUPPLEMENTARY FIGURE S2 Receiver operating characteristic (ROC) curves of 7515 candidate genes. **(A–G)** ROC analysis of seven candidate genes

SUPPLEMENTARY FIGURE S3 GO enrichment analysis of differential genes in B cell518 subpopulations

SUPPLEMENTARY FIGURE S4 GO enrichment analysis of differential genes in Monocyte521 subpopulations

SUPPLEMENTARY FIGURE S5 GO enrichment analysis of differential 523 genes in NK cell524 subpopulations

SUPPLEMENTARY FIGURE S6 GO enrichment analysis of differential genes in T cell subpopulation

SUPPLEMENTARY TABLE S1-2 Up and down-regulated genes common to sepsis and MetS

SUPPLEMENTARY TABLE S3-4 Results of GO enrichment analysis of up- and down regulated genes common to sepsis and MetS

SUPPLEMENTARY TABLE S5 The top ten marker genes for the 21 clusters

SUPPLEMENTARY TABLE S6-9 Results of differential analysis of monocyte, NK cell, Tcell and B cell subsets in sepsis single cell data
