# Supplementary figures and images for: Identification of new co-diagnostic genes for sepsis and metabolic syndrome using single-cell data analysis and machine learning algorithms
Source: Front Genet. 2023 Mar 16;14:1129476. doi: 10.3389/fgene.2023.1129476 (PMC10060809; doi:10.3389/fgene.2023.1129476)

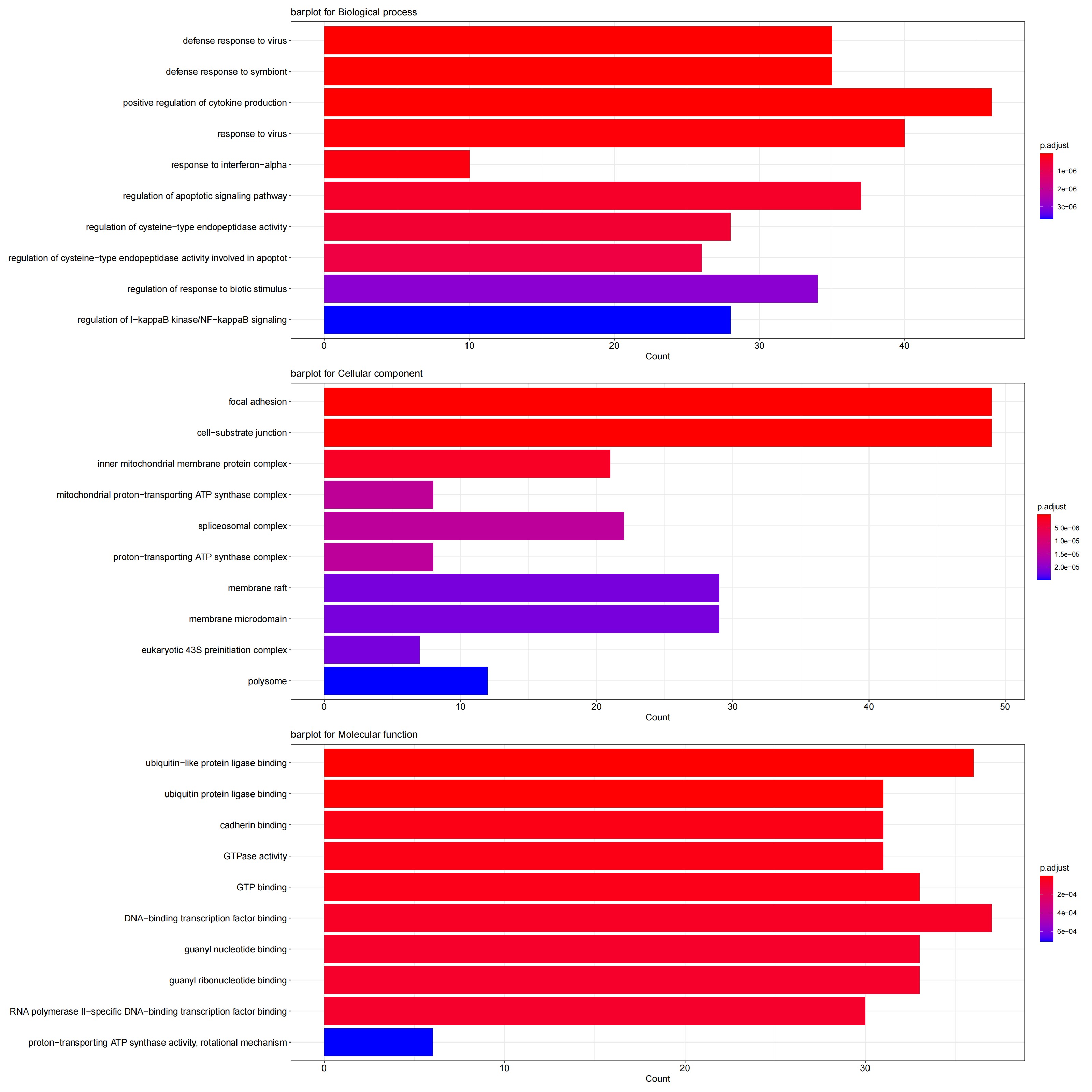

Supplement: Supplementary file 4 [file Image6.TIF]

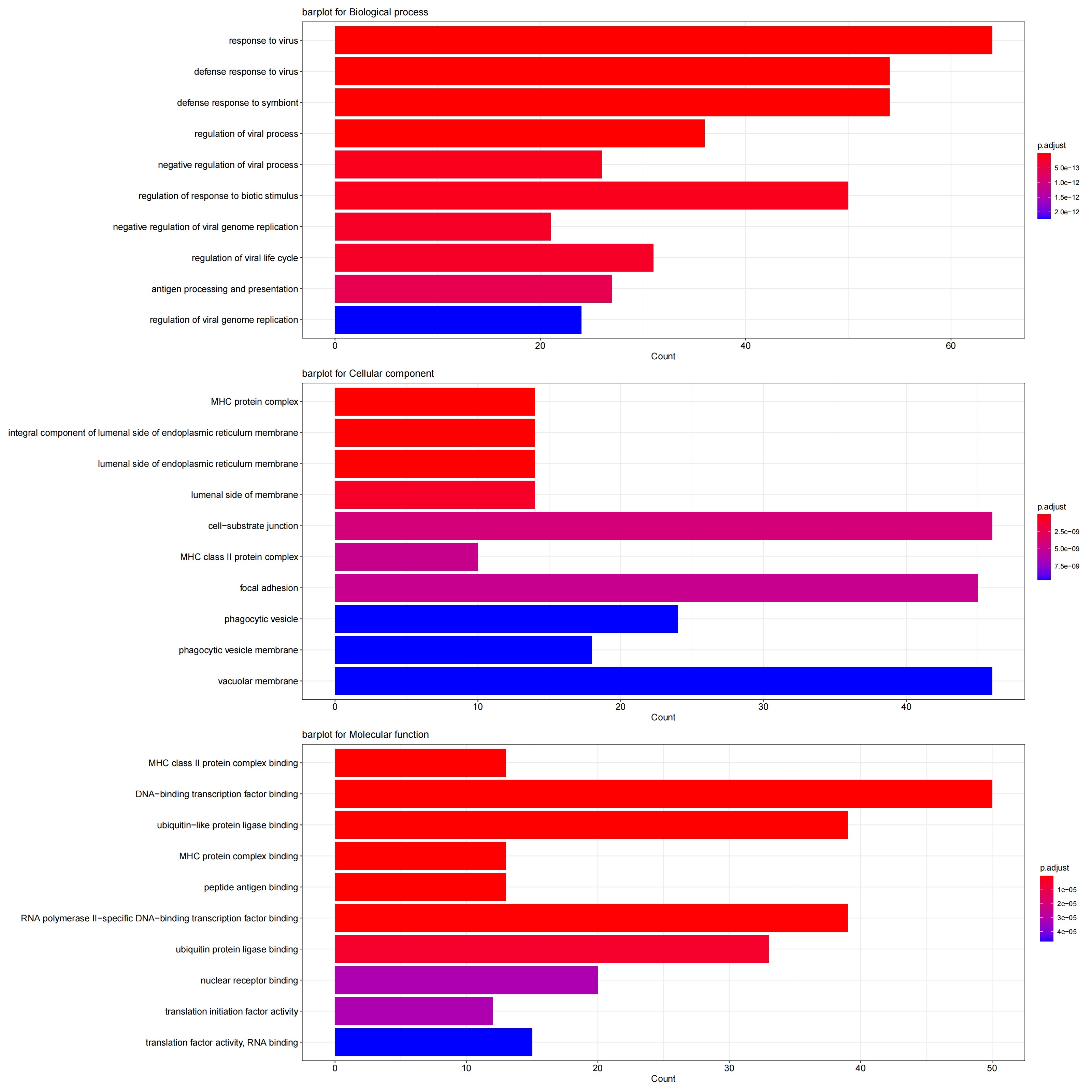

Supplement: Supplementary file 5 [file Image3.TIF]

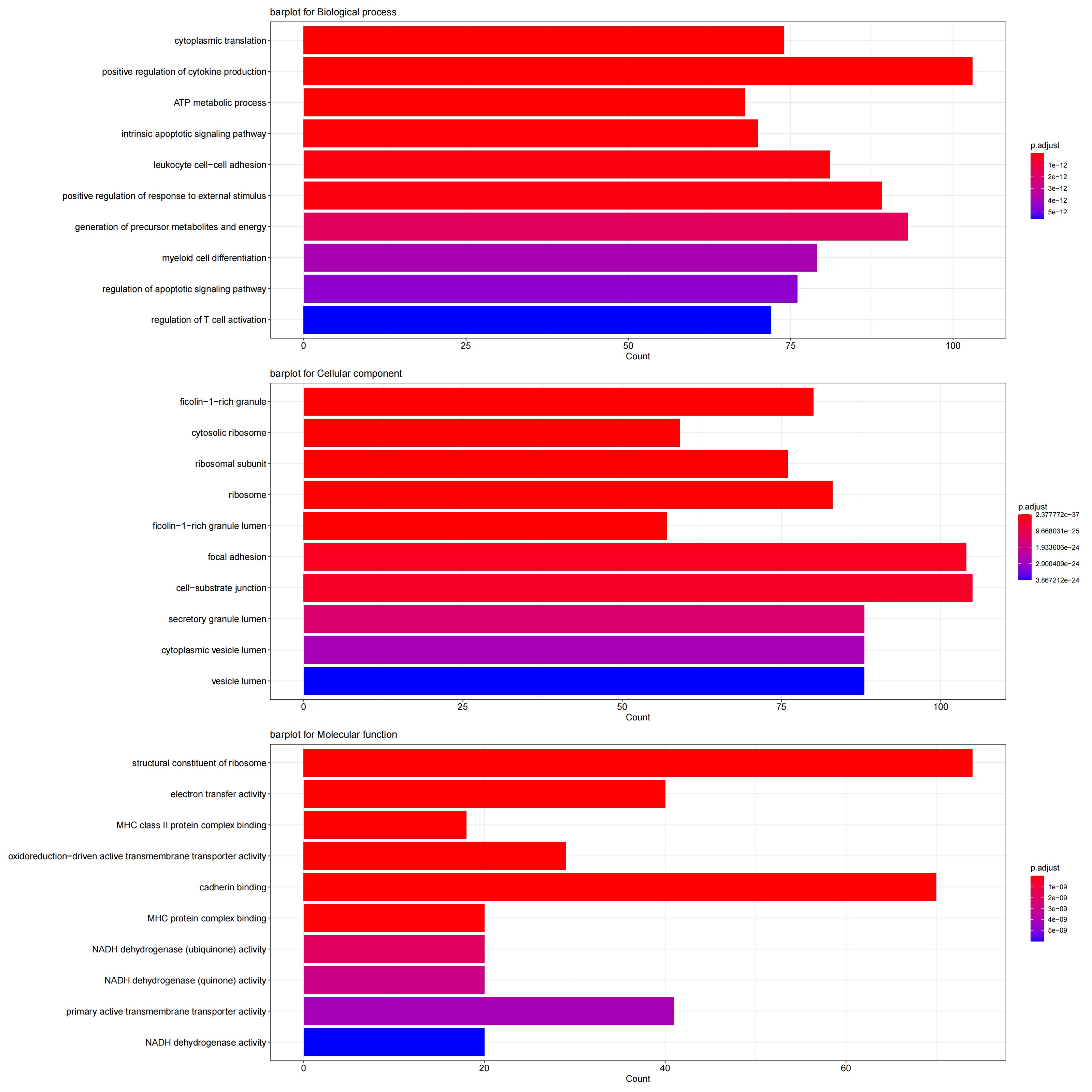

Supplement: Supplementary file 6 [file Image4.TIF]

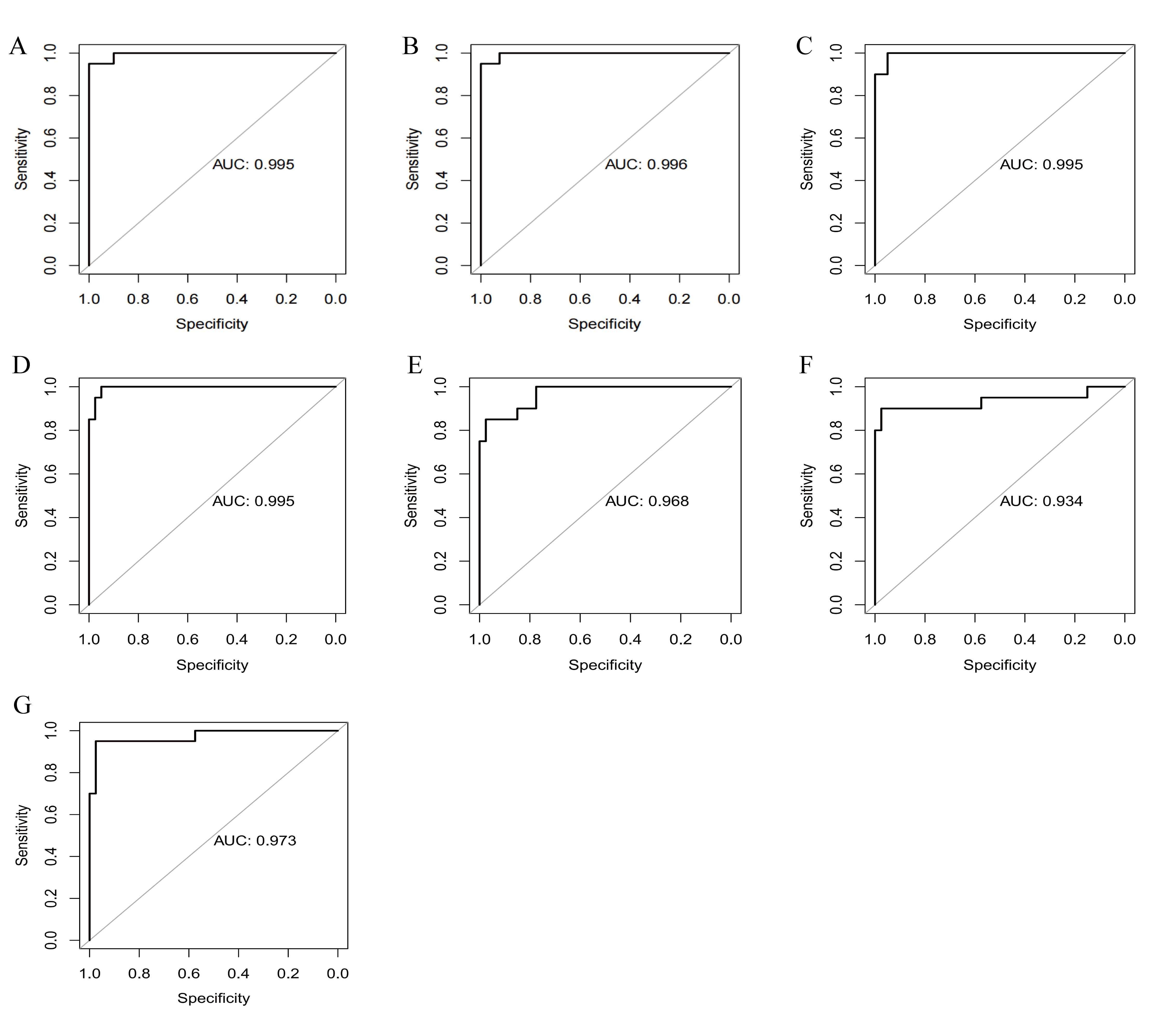

Supplement: Supplementary file 7 [file Image2.TIF]

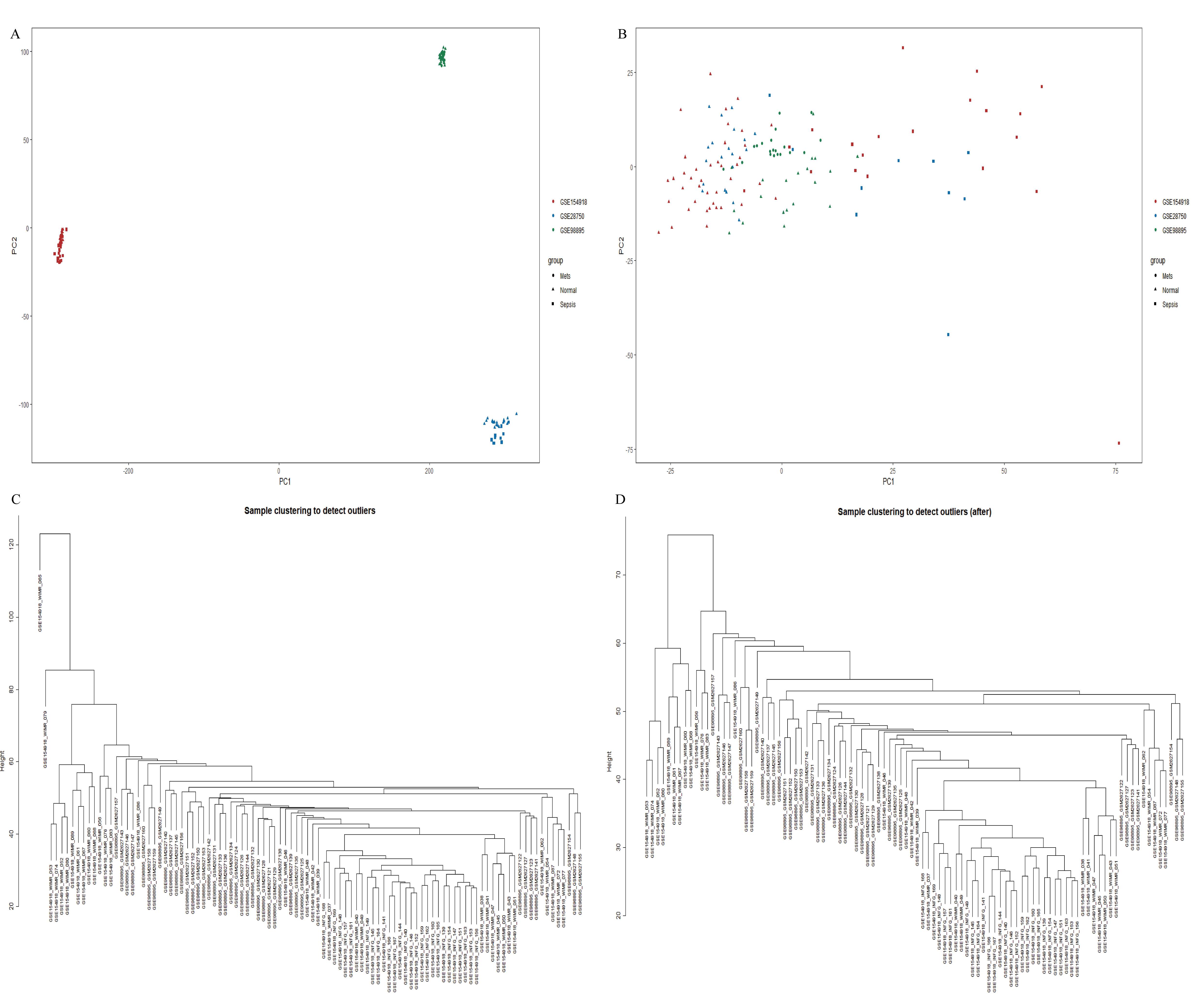

Supplement: Supplementary file 9 [file Image1.TIF]
